# Gases in Sea Water

**Published:** 1847-01

**Authors:** 


*Gases in sea water*—M. Lewy states that sea water holds only half as much gaseous matter as river water, and that the quantity varies according to the hours of the day. Thus he found in the waters of the ocean the following proportions:

| | Morning. | Evening. |
|---|---|---|
| Carbonic acid, - - | 3.4 | 2.9 |
| Oxygen, - - - | 5.4 | .6 |
| Nitrogen, - - - | 11. | 11.6 |
| | 19.8 | 20.5 |

